# From tradition to transformation: evolving models of care in clinical genetics

**DOI:** 10.1097/MOP.0000000000001502

**Published:** 2025-08-20

**Authors:** Helen Curd, Anita Gorrie, Andrew Paul Fennell

**Affiliations:** a Monash Genetics, Monash Health; b Department of Paediatrics, Monash University, Melbourne, Victoria, Australia

**Keywords:** clinical genetics, genetics workforce, genomics MDT, genomics models of care, mainstreaming genomics

## Abstract

**Purpose of review:**

The integration of genomics into mainstream healthcare is transforming clinical genetics into a foundational component of modern medicine. This review explores the evolution of clinical genetics service delivery, highlighting evolving models of care designed to meet rising demand, improve access, and ensure equitable, patient-centered genomic care.

**Recent findings:**

Key models of care discussed include multidisciplinary team approaches, embedded genetic counselors, advanced practice providers, upskilled non-genetics specialists, laboratory-based genetics clinicians, primary care providers of genetic healthcare and automated/patient-directed models. Educational needs, funding and adjuncts such as genetic assistants, collaborative telegenetics, and digital tools are also discussed for their role in supporting sustainable implementation.

**Summary:**

We recommend health organizations develop a roadmap for genomic medicine through creation of a genomic medicine governance framework, assessment of workforce capacity, definition of patient cohorts, and reviewing their infrastructure readiness. No single model of care is suitable for every context. By clearly defining needs, acknowledging limitations, and identifying potential risks, organizations can select the most appropriate models to address both current and future requirements. As genomics becomes increasingly embedded in routine care, we believe a coordinated, evidence-based approach is essential to ensure well tolerated, effective, accessible, equitable, and sustainable delivery of genomic medicine across diverse healthcare settings.

## INTRODUCTION

Since its inception in the mid-twentieth century, genetic healthcare has been delivered through centralized, hospital-based models of care (MoCs) delivered by clinical geneticists and, later, genetic counselors. These specialized clinical genetics services (CGS), often within academic or public health institutions, rely on labor-intensive, referral-based care that is not self-supporting (Figure S1) [1–4].

Early CGS models acted as gatekeepers to a narrow repertoire of slow, high-cost tests, relying heavily on clinical acumen and detailed family histories [5,6]. Advances in genetic testing technology, particularly the advent of NGS has enabled simultaneous analysis of multiple genes with faster turnaround, lower costs, and deeper insights into disease mechanisms and therapeutic targets [7,8]. Due to limited, highly specialized staffing, traditional CGS MoCs are typified by low accessibility, scalability and cost efficiency. While effective, they have become increasingly strained by the rising demand unlocked by the broader clinical applications of NGS [4].

Today, comprehensive genomic technologies – including NGS panels, exome sequencing, genome sequencing, and other multiomic approaches – support diagnosis of both rare and common disorders, guide precision therapeutic decisions, facilitate pharmacogenomic and carrier screening, and inform reproductive planning [3,4,9]. Genomic testing is now integral to routine care across many specialties [10,11], yet global resources remain insufficient to meet demand reinforcing the need for scalable, integrated approaches [4,12–14]. These pressures have prompted the development of innovative MoCs to aid ‘mainstreaming’ of genetics, embedding genetic testing into routine clinical pathways [15^▪▪^].

Literature describes a variety of modified traditional MoCs, evolving MoCs and associated needs. These approaches often overlap, and no single model fits all contexts. This review summarizes the MoCs that will shape the future of genomic medicine. 

**Box 1:**
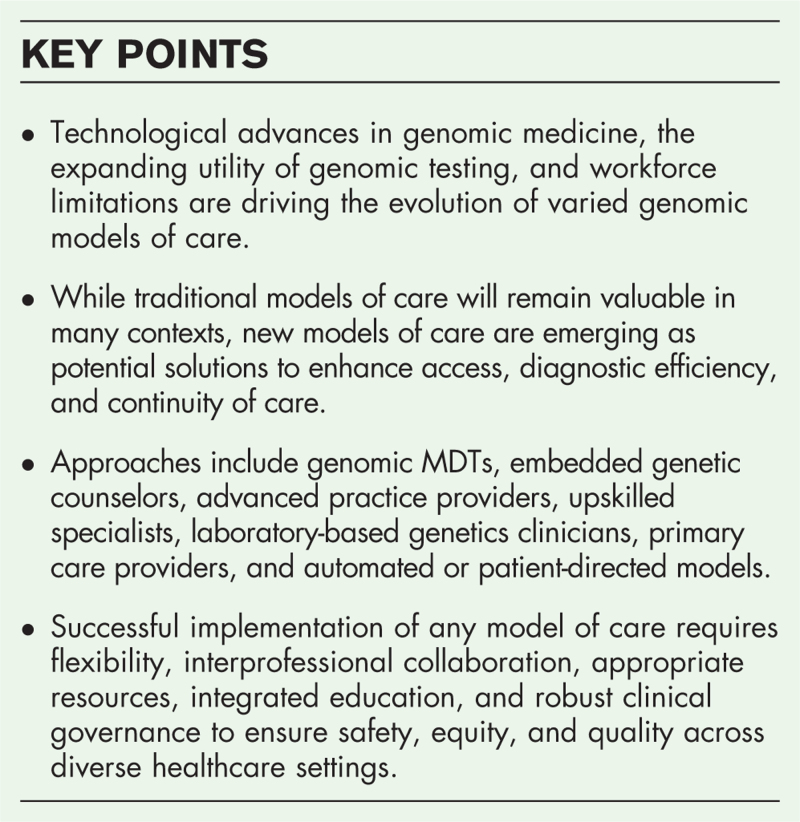
no caption available

## TRADITIONAL MODELS OF CARE

**FIGURE 1:**
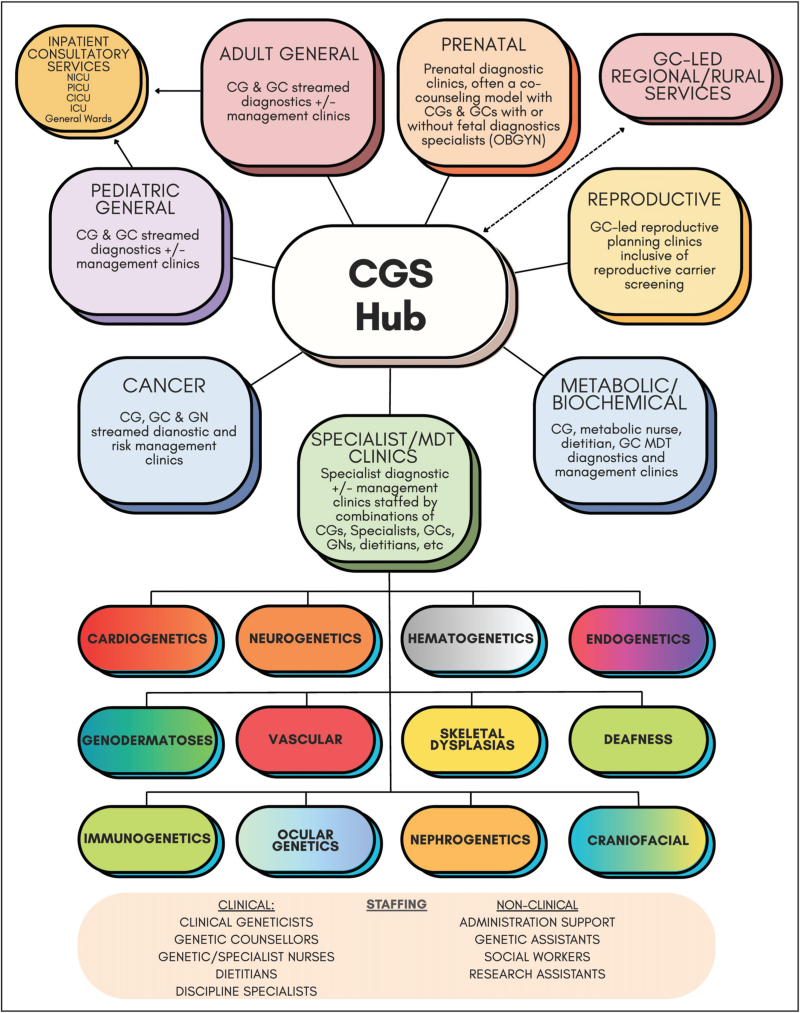
Components of a traditional centralized clinical genetics service. Solid lines indicate formal reporting or oversight relationships while the dotted line indicates a professional relationship. Human resources common to CGS are detailed under staffing. CG, clinical geneticist; CICU, cardiac ICU; GC, genetic counselor; GN, genetic nurse; NICU, neonatal ICU; PICU, pediatric ICU.

Traditional CGS have long incorporated multiple MoCs based on the needs of their population geography, specialist services and service demands, often built around a hub-and-spoke system (Fig. 1). The hub-and-spoke system organizes comprehensive CGS around a central tertiary CGS hub, supporting peripheral spoke sites tailored to local needs [16]. Many states in our jurisdiction, Australia organize their CGS as hub-and-spoke systems to balance the needs of metropolitan, regional and remote communities. In these cases, the hub is usually responsible for governance, management, service delivery and allocation of funding for genetic testing within their catchment. Regular components include core clinical geneticist and genetic counselor services, multidisciplinary genetics clinics serviced in tandem with specialists [17,18], outreach clinics where hub-based genetic specialists serve multiple primary care sites [19,20], and genetic counselor led services which are often independently run but typically have limited clinical geneticist or tertiary-center support. The latter are usually located in regional and rural areas [21].

## EVOLVING MODELS OF GENOMIC SERVICE DELIVERY

Recent literature reveals multiple MoCs to support greater access to genomic medicine. The structure of these newer MoCs detailed below, vary depending on local service needs, staffing, infrastructure, and funding. Some models involve clinical genetics professionals throughout the process, while others rely on non-genetics clinicians to lead, with CGS involvement when required [15^▪▪^,^22^]. There is no one-size-fits-all model, and the MoCs may require adaptation over time as needs change.

The key MoC features are outlined in Table 1 and components summarized in Table 2.

**Table 1:** Evolving models of care key features

Model name	Key features	Scope of practice
Genomic MDT	∘ Genetics health professionals and specialist clinicians meet for case discussions pertaining to: ∘ prospective genomics-suitable cases ∘ test selection ∘ funding approvals ∘ results discussion ∘ precision therapy options ∘ research opportunities ∘ case management issues	∘ Case review ∘ Test eligibility ∘ Result interpretation ∘ Clinical correlation ∘ Treatment and management planning ∘ Education and mentorship
Embedded GC	∘ GCs embedded within specialist clinics to work with specialty clinicians to: ∘ Educate specialists in clinical genetics process, logistical and ethical issues ∘ Identify genomics-suitable cases ∘ Offer in-clinic genetic counseling ∘ Facilitate liaison with CGs ∘ Testing logistical support	∘ Pre/post-test counseling ∘ Test coordination ∘ Cascade testing ∘ Reproductive information and planning ∘ Support ∘ Education
Advanced Practice Provider	∘ Nurses and physician assistants/associates deliver targeted genomic care after completing specialized training fellowship	∘ Scope dependent on training pathway and jurisdictional regulation but can include: ∘ Consent and counseling ∘ Ordering tests ∘ Results follow-up under supervision
Upskilled Specialist	∘ Specialist clinicians are equipped with targeted education and decision-support tools. ∘ CGS may provide backend support and follow up complex cases and cascade testing. ∘ Governance frameworks ensure quality assurance, credentialling, and appropriate escalation pathways for complex cases.	∘ Identification of eligible patients ∘ Consent to genomic testing ∘ Test ordering ∘ Simple result interpretation ∘ Patient treatment and management.
Laboratory-Based Genetics Clinician	∘ Pretest review by GCs/CGs to assess test appropriateness ∘ Requesting clinician liaison ∘ Improved test utilization management, reducing unnecessary costs, and enhanced diagnostic yield	∘ Modification or cancellation of mis-ordered or redundant tests ∘ Test/variant interpretation ∘ Result reporting ∘ Clinician support and education ∘ Development of patient resources
Primary Care Provider	∘ PCPs are upskilled or supported by embedded GCs to deliver low complexity genomic care, e.g. risk assessments, reproductive genetic carrier screening, pharmacogenomics, noninvasive prenatal screening	∘ Basic genomic testing and screening referrals ∘ Consent and counseling for same ∘ Patient management
Automated/Patient-Directed	∘ Individuals initiate genomic testing and counseling through digital platforms, often supported by chatbots, algorithms, and telehealth systems.	∘ Initial risk assessment via algorithms ∘ Pre-test education and consent ∘ Test ordering ∘ Post-test counseling (automated or human-assisted)

CG, clinical geneticist; CGS, clinical genetics service; GC, genetic counselor; PCP, primary care provider.

**Table 2:** Evolving models of care components

Model name	Key personnel	Supporting personnel	Setting	Strengths	Limitations
Genomic MDT	∘ CGs ∘ Specialists	∘ GCs ∘ GNs ∘ APPs ∘ Genomics lab personnel ∘ Other Clinicians	∘ Medical Centers	∘ Collaborative decision-making ∘ Comprehensive expertise ∘ Scalable	∘ Resource-intensive ∘ Scheduling complexity
Embedded GC	∘ GCs ∘ Specialty Clinicians	∘ CGs	∘ Specialist Clinics	∘ Integrated care ∘ Timely access to genetics expertise ∘ Adaptable to standalone services such as fertility medicine and antenatal care providers ∘ Scalable	∘ Dependent on the availability of trained GCs
Advanced Practice Provider	∘ Nurse Practitioners ∘ Physician Assistants	∘ CGs ∘ GCs	∘ Medical Centers	∘ Expands workforce capacity ∘ Accessible care ∘ Scalable	∘ Limited scope of practice ∘ Requires additional training and oversight
Upskilled Specialist	∘ Specialty Clinicians	∘ CGs ∘ GCs	∘ Specialist Clinics	∘ Leverages existing patient relationships ∘ Point of care testing reduces CGS referrals ∘ Efficient ∘ Scalable	∘ Variable expertise ∘ Risk of inappropriate ordering ∘ Oversight/governance required ∘ Guidelines/decision aids necessary ∘ Ongoing education requirements,
Laboratory-Based Genetics Clinician	∘ CGs ∘ GCs	∘ Clinical Laboratory Geneticists ∘ Genetic pathologists ∘ Laboratory scientists ∘ Genomics curators	∘ Genetic Testing Laboratories	∘ High technical expertise ∘ Reduced inappropriate ordering/wastage ∘ Rapid result interpretation ∘ Clinically relevant reporting	∘ Limited direct patient interaction ∘ Additional cost for genomics labs
Primary Care Provider	∘ Primary Care Physicians	∘ GCs	∘ Primary Care Clinics	∘ Broad reach ∘ Early identification of genetic risks ∘ Facilitates low complexity/low risk testing in the community ∘ Scalable	∘ Limited depth of expertise ∘ Training burden ∘ Dependent on availability of trained GCs ∘ No or limited CG support
Automated/Patient-Directed	∘ Software Developers ∘ CGs ∘ GCs ∘ Digital Health Administrators	∘ Legal and compliance officers	∘ Online	∘ Increased accessibility ∘ Cost-effective ∘ Reduced clinician burden ∘ Scalable	∘ Upfront investment, ∘ Limited emotional support and nuance ∘ Risk of misinterpretation ∘ Privacy concerns ∘ Unsuitable for complex or rare cases

APP, advanced practice provider; CG, clinical geneticist; CGS, clinical genetics service; GC, genetic counselor; GN, genetic nurse.

## GENOMIC MULTIDISCIPLINARY TEAM MODEL

The multidisciplinary team (MDT) meeting model has become a cornerstone of integrating genomics into routine clinical care [23,24]. Genomic MDTs bring together health professionals such as physicians, surgeons, nurses, pathologists, radiologists, pharmacists, primary care physicians, etc., together with genetics specialists such as clinical geneticists, genetic counselors, clinical laboratory geneticists, and genetic nurses [25]. Genomic MDT meetings enable collaborative case discussions, ensuring informed clinical decision-making and appropriate use of genomic testing. MDT meetings have been shown to significantly enhance diagnostic yield in genomic medicine and they enhance the accuracy of variant interpretation through review of variants of uncertain significance and genotype-phenotype correlations [26,27,28^▪▪^,^29^]. They also facilitate precision clinical management planning, especially in urgent settings like neonatal ICUs [24,30,31].

Human resource, funding and time constraints, as well as the need for institutional support and governance, are consistently reported challenges [23,28^▪▪^,32^▪^]. The literature positions MDTs as a practical, scalable, and clinically impactful model for delivering genomic medicine, enhancing diagnostic precision, fostering interdisciplinary collaboration, promoting genomic education and outreach [23] and facilitating responsible, equitable integration of genomics into healthcare [33].

## EMBEDDED GENETIC COUNSELOR MODEL

This model integrates genetic counselors directly into specialty clinics. It enables timely, coordinated genomic care and reduces reliance on external referrals to a centralized CGS hub. This approach streamlines access to testing, minimizes delays, and prevents bottlenecks in overstretched CGS [22]. Embedded GCs can contribute to MDTs, support clinical decision-making, assist with variant interpretation, and provide holistic patient care from consent to results disclosure, that considers both medical and familial needs.

Reported challenges include genetic counselor workforce shortages, the need for managerial support, and feelings of isolation by genetic counselors [22]. Clinical geneticist clinical supervision requirements must be considered in this model; a challenge with workforce shortages in many countries [34]. Despite these barriers, embedding genetic counselors is increasingly recognized as a cost-effective strategy that enhances patient outcomes, improves access, reduces CGS referrals, and supports the sustainable integration of genomics into mainstream medicine [27,35,36].

## ADVANCED PRACTICE PROVIDER MODEL

Kinney and colleagues recently described their experience integrating advanced practice providers (APPs) at the Medical College of Wisconsin [37^▪▪^]. This innovative response to critical workforce shortages of clinical geneticists and, to a lesser extent, genetic counselors shows how non-genetics clinicians can be upskilled and incorporated into genomic medicine. In their context, Clinical Genetics APPs (CGAPPs) are nurse practitioners and physician assistants/associates who undergo condensed resident competency training. The aim is for CGAPPs to reach a level of autonomous practice to realize the efficiency gains critical to this model.

While this is only a single-center experience and operates within the traditional CGS structure, their significantly increased clinical capacity, improved patient satisfaction scores and cost-neutral implementation, point to a model that warrants consideration at similarly structured and/or resource-limited centers. There are formalized training pathways at some US centers, however, there remains a lack of standardized certification or credentialing to support a consistent CGAPP scope of practice.

## UPSKILLED SPECIALIST MODEL

The Upskilled Specialist Model is a collaborative MoC in which non-genetics medical specialists – such as pediatricians, oncologists, neurologists, etc. – are trained and supported to initiate genomic testing within their scope of practice. It aims to mainstream genomic medicine by embedding testing into routine clinical workflows, particularly where genomic information can directly inform diagnosis, prognosis, or treatment [38]. This MoC enables broader access to genomic medicine with genetic specialist expertise focusing on complex or undiagnosed cases [15^▪▪^].

This model has demonstrated success in oncology [38,39] and is increasingly being explored in pediatric settings [40]. It improves patient access to timely testing, enhances clinician confidence, and significantly reduces the burden on CGS [39].

To ensure safe and effective implementation, this MoC requires careful credentialing, governance and specialty-specific safeguards such as clear guidelines, eligibility criteria and resource support [24]. However, the lack of targeted, structured education and aligned credentialing pathways remain major barriers. Medical boards and postgraduate medical colleges are well positioned to take a leading role in establishing minimum competencies and structured pathways for specialty-specific genomics credentialing [15^▪▪^].

Another key challenge is the time burden on non-genetics clinicians when incorporating genomics into routine appointments [40]. Additional challenges include maintaining consistent knowledge retention among clinicians, ensuring high-quality informed consent, and addressing disparities across different healthcare settings [41].

## LABORATORY-BASED GENETIC CLINICIAN MODEL

This model involves embedding genetic counselors and clinical geneticists within genomic laboratories. The primary aim is to ensure that the most appropriate, cost-effective, and clinically relevant tests are requested, however laboratory-based clinician roles are expanding to include variant interpretation, clinically-relevant reporting and clinician education, both in germline and somatic workflows [42–44].

A recent scoping review synthesized findings from multiple studies and confirmed that laboratory-based genetic counselors significantly reduce inappropriate testing and positively influence non-genetics providers’ ordering practices [45]. For example, Suarez *et al.* [46] reported that 20% of test orders were modified or cancelled after genetic counselor review, while other studies demonstrated improved cost-effectiveness through test utilization management [47–49].

## PRIMARY CARE PROVIDER MODEL

Primary care providers (PCPs) are well positioned to deliver precision health in routine care. Emery and Hayflick [50] emphasized this potential over two decades ago, noting PCPs’ role in genetic risk assessment, screening and pharmacogenetics. Despite growing expectations for PCPs to offer services like carrier screening and non-invasive prenatal testing, barriers such as limited knowledge and system-level support persist [51^▪^]. Embedding genetic counselors into primary care teams, as proposed by Pan *et al.* [52], offers a collaborative approach to improve access, continuity, and alignment of genomic services with patients’ ongoing healthcare needs. This integration supports proactive, prevention-focused care and long-term guidance as their genetic needs evolve [51▪,52]. Massart *et al.* [53] provide a practical example through a multidisciplinary clinic where PCPs, pharmacists, and genetic counselors deliver integrated genomic services.

## AUTOMATED/PATIENT-DIRECTED MODELS

Automated and patient-directed models are reshaping genetic service delivery by enabling individuals to initiate testing and counseling through digital platforms. Mittendorf *et al.* [54] demonstrated that electronic family history tools, integrated with electronic health records, can efficiently identify patients at risk for hereditary cancer syndromes and streamline referrals. Similarly, Kaphingst *et al.* [55] found that chatbot-based pretest education was equivalent to standard genetic counseling in terms of service uptake and testing completion, supporting the scalability of such models. The ‘Genetics Navigator’ is another recently developed digital tool that is designed to support the full spectrum of genetic services [56]. These innovations offer accessible, efficient solutions to meet growing demand and alleviate pressure on the genetic workforce while offering opportunities for implementation at population scale for carrier screening and pharmacogenomics.

## FUTURE CONSIDERATIONS FOR CELL AND GENE THERAPIES

Cell and gene therapies (CGT) are in rapid development and represent a significant opportunity to impact care for those with genetic diseases. However, there are many challenges that necessitate models of care tailored to the specific geographical and jurisdictional context [57]. CGS are well positioned to be core components of a CGT model working with clinical pharmacology, immunology and specialists to molecularly diagnose, identify therapeutic opportunities and deliver precision care. These CGT models could be built as hub-and-spoke systems as highlighted by recent publications [57,58] but an optimal MoC has yet to be determined [59].

## EDUCATION

The literature consistently highlights that integrating genomics into mainstream clinical care requires widespread education of non-genetics healthcare professionals to improve genomic literacy and ensure clinicians can confidently engage in test selection, consent, result interpretation and appropriate follow-up [24]. Key needs include tailored, evidence-based education [60], scalable e-learning [61], and robust evaluation of education programs [62].

Research indicates ongoing gaps in genetics and genomic medicine education among healthcare providers and recommends integrating comprehensive instruction and clinical exposure into medical and nursing school curricula [63–68]. The Association of Professors of Human and Medical Genetics (APHMG) and the Undergraduate Training in Genomics (UTRIG) working group have previously published medical school core competencies and integrable educational modules, respectively [68]. However, obstacles remain, including limited faculty expertise, the absence of established contemporary standards, and competing educational priorities. Addressing these challenges requires policy support, dedicated funding, and institutional commitment to genomics education [63].

For practicing clinicians, ongoing education in genomics must be accessible, relevant, and adaptable to busy clinical workflows [60,69]. Challenges for non-genetics medical specialists include time constraints, competing priorities, lack of structured learning opportunities, and variability of baseline knowledge [70,71]. Specialists emphasize the importance of tailored education, peer interactions that contextualize genomics knowledge, and experiential learning to build confidence and skills [70]. Continuing professional development through online modules, webinars, and microcredentialing can provide flexible specialty-specific learning opportunities [69,72]. Embedding genomics decision-support tools within electronic medical records can offer real-time guidance, while interdisciplinary collaboration with genetic counselors and clinical geneticists can foster informal, case-based learning.

The Competency-based Online Genomic Medicine Training set of online training modules represents an innovative approach to cooperatively deliver educational programs in this space [69]. Stellacci *et al.* [61] demonstrated the effectiveness of a problem-based e-learning course in oncogenomics, which significantly improved knowledge across disciplines. These demonstrate that scalable online genomics education, aligned with core competencies can effectively support continuing education. Professional standards bodies and specialty societies also play a key role in defining competency frameworks and disseminating best practices, helping to ensure that genomic literacy continues to evolve alongside clinical care [73,74].

## FUNDING AND SUSTAINABILITY

While genomic MoCs continue to evolve, their financial sustainability is increasingly recognized as a critical challenge across healthcare systems [32^▪^,^75^]. CGS and genomic programs globally struggle to generate sufficient revenue to cover operational costs, raising concerns about long-term viability [32^▪^]. CGS are typically considered a net expenditure within health service budgets; nonetheless, assessing their whole-of-service cost-effectiveness and associated economic and health benefits is complex, as gains manifest across both the health service and the broader population [76,77]. Substantial evidence demonstrates that genomic testing can lead to cost savings and better patient outcomes [78–82].

To date, mainstreaming genomic MoCs have generally been implemented in a research capacity as proof-of-concept initiatives. While the development of these MoCs appears to have been primarily driven by objectives related to cost saving/containment and addressing genetics workforce constraints, data to support their ongoing cost-effectiveness are largely absent. It is generally anticipated that these MoCs will not incur higher costs than existing CGS MoCs; however, broader implementation may result in increased demand and higher clinical throughput, ultimately leading to greater expenditures for clinical and pathological services. Significant cost savings are expected at the wider health system level, stemming from earlier interventions, reductions in inappropriate therapies, fewer unnecessary investigations, and decreased admission rates or lengths of stay. Cost savings are also expected at the population level, through prevention, improved health outcomes, reduced reliance on disability supports and safety nets, and enhanced economic productivity. However, these potential benefits are complex and remain to be thoroughly quantified across the vast array of indications for genomic diagnostics.

In the United States, access to CGS and genetic testing varies by jurisdiction based on healthcare systems, insurance coverage, and the availability of financial assistance programs and charitable initiatives [83,84]. The recent introduction of Current Procedural Terminology code 96041 enables billing for non–face-to-face genetic counseling services, expanding access and better reflecting the full scope of genetic counselor work [85]. However, challenges remain, particularly around state licensure, payer reimbursement and recognition by Medicare and Medicaid [85,86]. These challenges complicate the design and implementation of newer MoCs that require clinical geneticist and genetic counselor input, especially where they do not provide billable direct patient care.

Australia has similar funding complexities with its mixed public and private healthcare system. The Medicare Benefits Schedule (MBS) funds private clinical geneticist outpatient services. However, applicable item numbers provide insufficient funding to run practices without significant gap fees for patients. Although genetic counselors are recognized health professionals, they currently lack access to MBS funding through an item number [87]. As such, private practice genetic counselors are fully patient paid, which affects the equity and sustainability of private services. MBS pathology item numbers provide funding for genomic tests covering specific indications but do not reimburse for the clinical time required to administer these tests. This combination of limited clinical funding, and many indications being ineligible for MBS-funded genomic testing, results in restricted, inequitable access to these services privately. Public services are constrained by limited staffing and diagnostics budgets, with activity-based funding (ABF) models that undervalue CGS resourcing requirements. The recent introduction of ABF funding for genetic counseling has the potential to improve public service sustainability, particularly if new MoCs are implemented.

Recent reviews by Unim *et al.* [9] and Garavito *et al.* [32^▪^] highlight systemic challenges in sustaining genomic programs worldwide. Most programs operate with public funding yet face limitations in cost-effectiveness and economic evaluation. High implementation costs – including education, training, consent, integration, and relatively high test costs – are barriers to system-wide adoption [32^▪^]. Short-term funding models risk undermining long-term sustainability, and many programs remain in pilot phases without durable financial or policy commitments. Underserved areas are often further impacted as financial sustainability of genetic services in these areas often hinges on leveraging alternative funding sources, such as charity programs or grants, to overcome barriers to access and ensure equitable care delivery [83].

These findings underscore the need for robust reimbursement structures, economic modeling, and legislative frameworks to support the integration of genomics into routine care. Addressing systemic funding gaps is essential to ensure equitable and enduring access to genomic services across diverse healthcare settings.

## MoC ADJUNCTS

Facilitators of sustainable implementation of genomic medicine that can enhance traditional and evolving genomics MoCs are described below and detailed in supplementary Table 1.

## GENETIC ASSISTANTS

Genetic assistants are clinically trained support staff who enhance genetic services by collecting family histories and performing administrative and coordination tasks that exceed typical administrative roles but do not require the expertise of a genetic counselor [88,89]. Their implementation has been shown to lead to sustainable increases in genetic counselor patient volumes and decreased clinical cost per patient [89,90].

## COLLABORATIVE TELEGENETICS

Telehealth is a mode of delivering genomics that expands access to genetic services, especially in underserved areas, reducing travel, cost and wait times [91,92]. Kubendran and colleagues [93] described a collaborative multidisciplinary telegenetics model which demonstrated improved service efficiency and patient satisfaction. Further recent studies noted patient satisfaction did not differ between in-person and telegenetic modes [94,95].

## TOOLS AND RESOURCES

Digital innovations are playing an increasingly vital role in supporting the integration of genomics into clinical care. Supplementary Table 2 outlines digital tools and platforms that enhance service delivery, education, and patient engagement throughout genomic medicine pathways. Governance of digital tools is essential to standardize care and ensure that digital innovations are implemented safely and effectively.

## DISCUSSION

As genomic MoCs evolve, so do the roles of genetics professionals. Clinical geneticists are increasingly taking on leadership roles in genomics implementation, championing genomic diagnostics stewardship, managing complex cases, delivering CGTs, and providing genomic laboratory clinical oversight. Genetic counselors responsibilities have broadened to include clinical specialization, laboratory liaison roles, genomics education and research, and they are increasingly embedded in MDTs [92,96].

**FIGURE 2:**
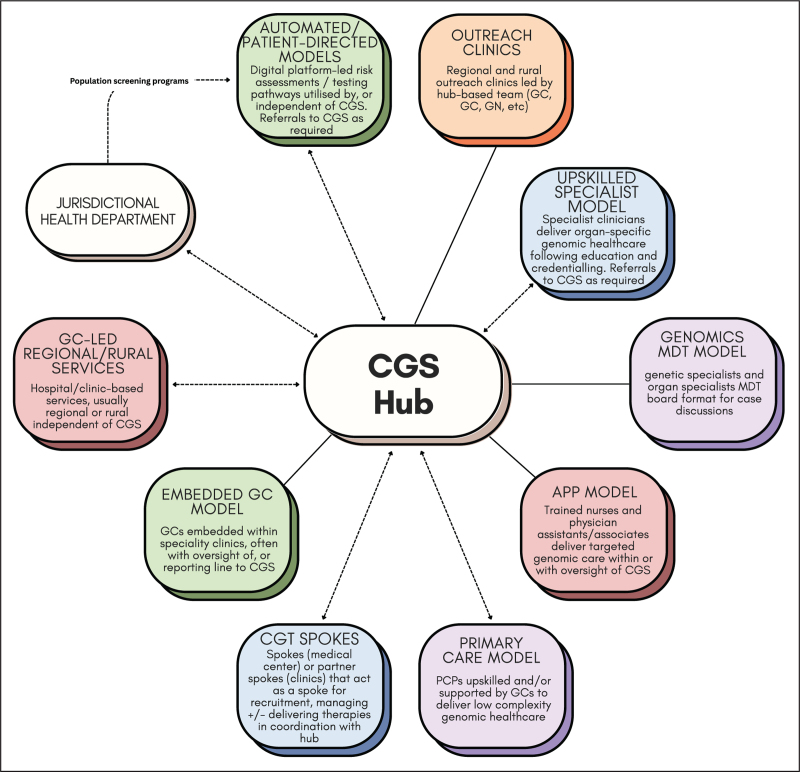
Conceptual clinical genetics service hub-and-spoke systems map integrating various contemporary models of care. CG, clinical geneticist; CGS, clinical genetics service; CGT, cell and gene therapies; GC, genetic counselor; GN, genetic nurse.

Although comprehensive CGS and traditional MoCs remain essential, alternative models that integrate genomics into mainstream clinical practice, supported by a CGS hub, offer significant advantages in many contexts (Fig. 2). These include increased capacity, improved access to testing and counseling, faster diagnoses, and enhanced access to personalized treatment and long-term care, ultimately improving patient outcomes across diverse settings [57,97].

Efficient, tailored education and training are critical to ensure competent and safe delivery of genomic services [29]. However, barriers such as low genomic literacy and limited confidence in result interpretation persist, underscoring the need for significant educational investment. Clear referral pathways for complex cases and access to MDT meetings may help mitigate these challenges.

Ethical, legal, and social considerations – including informed consent, data privacy, insurance implications, incidental findings, and familial implications – must be carefully managed [98]. Ensuring equitable access and addressing skepticism about genomics’ clinical utility are also critical [97]. Interdisciplinary collaboration among genetics and non-genetics clinicians, policymakers, medical colleges, research institutes, and patient advocates is vital to overcome these challenges.

Funding genomic care presents a substantial challenge. Although the authors are not health economists, it is evident that the cost and sustainability of genomic services are shaped by diverse, system-dependent funding models and infrastructure that vary significantly by region.

To implement genomic medicine effectively into routine clinical care, a whole-of-system approach is recommended at national, regional and healthcare service levels. We believe services should set a roadmap for genomic medicine [99]; develop a future-fit genomic medicine governance framework, assess workforce capacity and skills, define patient cohorts, and assess their infrastructure readiness. Through defining needs, recognizing limitations and identifying risks, the appropriate models to meet current and future needs can be chosen. Sustainable implementation of new MoCs depends on strong governance, a clear understanding of your population, strategic funding decisions and infrastructure investment, with strong executive support.

Robust evaluations of these models are needed. Comparative and longitudinal studies can identify models that balance genomics expertise, accessibility, scalability, cost efficiency, systems integration, research access, and patient outcomes across a range of service needs.

## CONCLUSION

As genomics becomes increasingly relevant across a wide spectrum of clinical contexts, novel MoCs are being considered to meet rising demand and to ensure timely, equitable, and patient-centered care. Innovative approaches – such as embedding genomic expertise within specialty services, fostering interdisciplinary collaboration, laboratory clinical support, and upskilling non-genetics professionals – are helping to bridge the gap between genetics specialists and mainstream care.

Mainstreaming genomics holds significant promise for improving the reach and effectiveness of genomic medicine; however, its success depends on rigorous governance, careful implementation, adequate support and funding structures, and ongoing workforce development. Patient and familial safety must be forefront when considering different MoCs. As the field of genomics continues to expand, collaborative, evidence-based approaches are vital to ensure well tolerated and equitable access and improve patient experience and health outcomes.

## Acknowledgements

*None.*

### Financial support and sponsorship

*None.*

### Conflicts of interest

*There are no conflicts of interest.*

## Supplementary Material

Supplemental Digital Content

## REFERENCES AND RECOMMENDED READING

Papers of particular interest, published within the annual period of review, have been highlighted as:

▪ of special interest
▪▪ of outstanding interest
