# Supplementary material for: From tradition to transformation: evolving models of care in clinical genetics
Source: Curr Opin Pediatr. 2025 Aug 20;37(6):538–49. doi: 10.1097/MOP.0000000000001502 (PMC12594115; doi:10.1097/MOP.0000000000001502)
Supplement: Supplemental Digital Content [file coped-37-538-s001.docx]

**Table of Contents**

[**Figure S1: Simplified workflow of a traditional core clinical genetics service** 2](#_Toc204351056)

[**Table S1: Adjuncts to genomics models of care** 3](#_Toc204351057)

[**Table S2: Tools and resources to support integration of genomics into clinical care** 3](#_Toc204351058)

[**References:** 4](#_Toc204351059)

# **Figure S1: Simplified workflow of a traditional core clinical genetics service**

This diagram highlights the traditional clinical genetics workflow from referral through triage, consultation, testing, diagnosis and onward referral or discharge.


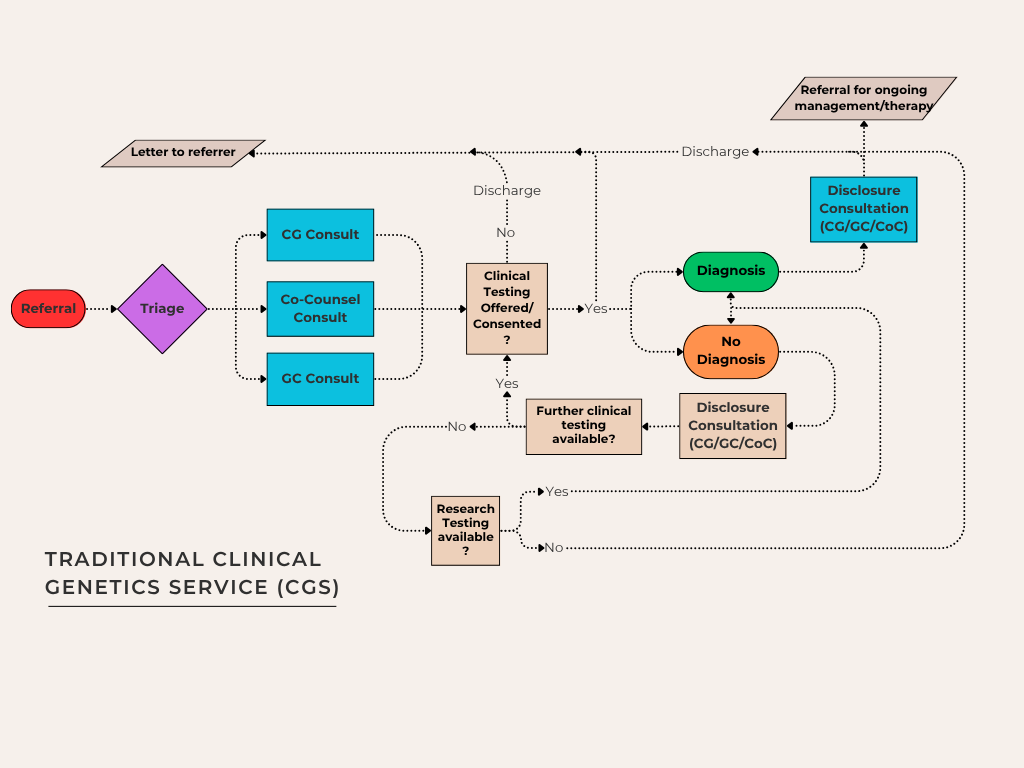


CG, clinical geneticist; GC, genetic counsellor; CoC, co-counselling.

# **Table S1: Adjuncts to genomics models of care**

| MOC Adjuncts | Key Features | Benefits | Challenges |
| --- | --- | --- | --- |
| Genetic Assistants | - Collect family histories - Gather documentation - Support testing logistics - Support cascade testing | - Frees GCs/CGs/Specialists to focus on clinical care and work to top of scope - Improves efficiency and patient throughput | - Requires clear role definition and training |
| Collaborative Telegenetics | - Genetic consults delivered via telehealth - Remote consultations - Example: Specialist and GC with patient in rural setting and CG delivering telehealth care as required | - Expanded access in underserved areas - Reduces travel, cost, wait times - Improved service efficiency - Patient satisfaction with mode | - Requires technological infrastructure - Inability to examine patients and read verbal cues if not in-person - Require close coordination across specialties |
| Tools & Resources | - Digital tools and platforms   (Further described in Table S2) | - Frees clinicians to perform other tasks - Efficient and cost effective - Easily accessible - Reduce risks | - Digital health governance requirements - Technological limitations - Digital literacy gaps - Need to ensure ongoing content quality and accuracy |

CG, clinical geneticist; GC, genetic counsellor

# **Table S2: Tools and resources to support integration of genomics into clinical care**

| Tool/Resource | Key Insights | Supporting Evidence |
| --- | --- | --- |
| Clinical Decision Supports | Digital tools enhance risk stratification, test selection, result interpretation, and management decision for clinicians.  Examples:   - **Pharmacogenomic-guided prescribing** - **Cancer Genetics risk stratification** - **Face2Gene** | - ^1^ Klein, et al. (2017) - ^2^ Johnson et al. (2024) |
| Professional Development Platforms | Online modules, webinars, and interactive tools support continuous learning and integration of genomics into practice. | - ^3^ Stoll (2018) - ^4^ Nisselle et al (2024) |
| Patient-Facing Digital Genomic Healthcare Tools | Tools support the full genomic testing journey, from education and consent to results and care planning. Critical components of the automated/patient-directed model of care.  Examples:   - **Gia** - **Genetics Adviser** - **Genomics ADvISER** - **Genetics Navigator** | - ^5^ Snir, et al. (2020) - ^6^ Clausen, et al. (2024) - ^7^ Bogue, et al. (2025) - ^8^ D’amours et al. (2024) - ^9^ Bombard et al. (2022) |
| Patient Decision Aids | Decision aids are specifically designed to help patients understand their options and make informed choices about specific clinical scenarios. | - ^10^ Freed, et al. (2021) |
| Clinical Genetics Data Management | Platforms enable secure storage of a variety of patient data, pedigree tracking, results, and/or family linkage, supporting clinical workflows and cascade testing. Limitations can include poor interoperability, lack of family linkage in EMRs, absence of integrated digital care pathways  Examples:   - **Trakgene** - **iGene** - **Progeny** - **Phenotips** - **EMR genetics/genomics modules** | - Clinical implementation reports - Platform documentation |

# **References:**

1. Klein ME, Parvez MM, Shin JG. Clinical Implementation of Pharmacogenomics for Personalized Precision Medicine: Barriers and Solutions. J Pharm Sci 2017;106:2368-79.

2. Johnson D, Del Fiol G, Kawamoto K, et al. Genetically guided precision medicine clinical decision support tools: a systematic review. J Am Med Inform Assoc 2024;31:1183-94.

3. Stoll K, Kubendran S, Cohen SA. The past, present and future of service delivery in genetic counseling: Keeping up in the era of precision medicine. Am J Med Genet C Semin Med Genet 2018;178:24-37.

4. Nisselle A, Terrill B, Janinski M, et al. Ensuring best practice in genomics education: A theory- and empirically informed evaluation framework. The American Journal of Human Genetics 2024;111:1497-507.

5. Snir M, Nazareth S, Simmons E, et al. Democratizing genomics: Leveraging software to make genetics an integral part of routine care. Am J Med Genet C Semin Med Genet 2021;187:14-27.

6. Clausen M, Krishnapillai S, Hirjikaka D, et al. Genetics Adviser: The development and usability testing of a new patient digital health application to support clinical genomic testing. Genet Med Open 2024;2:101814.

7. Bogue D, Douglas C, Miller E, Tatton-Brown K, Edi-Osagie N. Mainstreaming genomics in paediatrics. Arch Dis Child Educ Pract Ed 2025.

8. Amours G, Clausen M, Luca S, et al. Genetics Navigator: protocol for a mixed methods randomized controlled trial evaluating a digital platform to deliver genomic services in Canadian pediatric and adult populations. BMJ Open 2024;14:e090084.

9. Bombard Y, Ginsburg GS, Sturm AC, Zhou AY, Lemke AA. Digital health-enabled genomics: Opportunities and challenges. The American Journal of Human Genetics 2022;109:1190-8.

10. Freed AS, Gruss I, McMullen CK, et al. A decision aid for additional findings in genomic sequencing: Development and pilot testing. Patient Educ Couns 2021;104:960-8.
